# Nonsurgical Elementary Rhinoplasty: A Volumetric Standardized Hyaluronic Acid Filling Technique in Five Steps

**DOI:** 10.1055/s-0045-1802317

**Published:** 2025-01-31

**Authors:** Luddi L. Oliveira, André Braz, Eliandre Palermo, Maria C.A. Issa

**Affiliations:** 1 Plastic Surgery, Private Clinic, Belo Horizonte, Minas Gerais, Brazil; 2 Dermatology, Private Clinic, Rio de Janeiro, Rio de Janeiro, Brazil; 3 Dermatology, Private Clinic, São Paulo, São Paulo, Brazil; 4 Department of Dermatology, Department of Internal Medicine, Fluminense Federal University, Rio de Janeiro, Brazil

**Keywords:** hyaluronic acid, nose, rhinoplasty, cosmetic techniques

## Abstract

Hyaluronic acid filling to correct the nose contour has gained popularity in recent years. Although many techniques are described about the amount of the product and steps to follow to obtain, practitioners still need clarification about the amount of product and steps to follow obtain natural and safe results. We aim to demonstrate a new technique with a systematic sequence that considers the anatomy and individual characteristics. The nonsurgical elementary rhinoplasty (NOSER) technique is performed in five steps using cannulas, a high G prime hyaluronic acid, and two injection entry points. The areas to be treated and the specific quantity of filler to be used are based on the assessment. Steps 1 to 3 are for the tip's projection, support and lift, and step 4 is for filling the radix area and straightening the dorsum. Step 5 is used for refinement and softening of the supratip. All five steps are done in small aliquots in the central area, at the deep plane. Through the procedure, it is possible to achieve an aesthetic improvement in the nose by widening the labial columellar angle, exposing the columella, projecting the tip, and aligning the dorsum (disguising the hump), even without performing all five steps. This technique could be a good and highly reproducible alternative for upgrading the nose contour.

## Introduction

The nose influences the balance of the face and contributes to an individual's expression. 1 Recently, minimally invasive procedures to correct the nose contour using hyaluronic acid (HA) have emerged as a good alternative. 2 Despite many techniques, practitioners need clarification about the amount of product and steps to follow to obtain natural, beautiful results without complications. Thus, this study aims to demonstrate a new technique applied in a systematic sequence, using cannulas for nose corrections according to the anatomical and individual characteristics.

### Indications

**Fig. 1:**
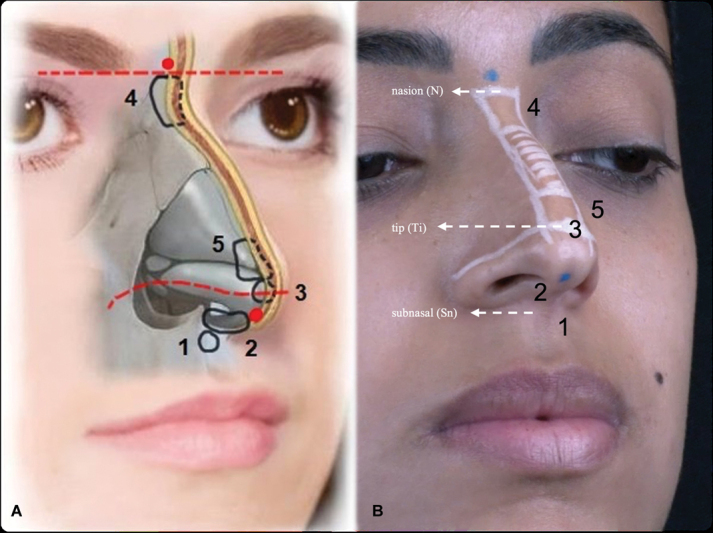
( **A** ) Schematic drawing of the nonsurgical elementary rhinoplasty (NOSER) technique and its steps. Entry points are marked in the glabella and at the end of the columella ( *red dots* ). Imaginary lines ( *dotted red lines* ) are drawn in the nasion (N) and the tip (Ti). The *areas outlined in black* represent the application steps. ( **B** ) The NOSER technique demonstrated in a live model. The dorsal hump is shaded in *white* . The entry point is indicated by the *blue dots* . Under the inferior entry point, step 1—anterior nasal spine; step 2—columella. Above the inferior entry point, step 3—tip; step 5—supratip break. Under the superior entry point, step 4—radix.

The nonsurgical elementary rhinoplasty (NOSER) technique is adequate for both women and men with a narrow nasal bridge width (distance between the dorsal aesthetic lines) up to 6 to 8 mm in women and up to 8 to 10 mm in men, nasal thin skin, dorsal hump (height <1.8–2cm), droopy tip (a nasal tip that points downward), and acute nasal labial angle. 3 The procedure is based on five steps and only two insertion sites, using a 22-/23-gauge cannula and a high G prime HA (high tissue lifting capacity). 4 The gel is applied in the central area of the nose and always at a deep level (supraperichondrial and supraperiosteal) due to the predominant location of the nasal arteries laterally and superficially (subcutaneous plane 5 ; Fig. 1A ).

### Patient's Preparation



**Video 1** Demonstration of the marks that should be made before the hyaluronic acid (HA) application.


Apply topical lidocaine 7% at the entry points 30 minutes before the procedure. Meanwhile, draw a rectangle to limit the upper, lower, and lateral boundaries of the dorsal hump, which should not be filled, touched, or detached. Thus, continue delimiting the nasal aesthetic dorsal lines that come from the orbital rim and go straight down to the nasal wings (two points of light of the nose), establishing the central part of the nose. On the nasion point (N), which cephalometrically determines the beginning of the nose length (above it is considered the glabella), an imaginary transversal line is drawn as a reference that should pass through the upper eyelid sulcus.

Another horizontal line is drawn between the nasal wings, merging at the center of the lateral crests of the inferior nasal lateral alar cartilages, where corresponds to the cephalometric point tip (Ti). This is a very important site where the patient's new nose projection will be and the nasal length ends. The last markings are the entry points: one in the glabella (just above the nasion) to fill the superior part of the nose and another in the columella (at the level of the upper part of the nostrils) to inject the inferior areas of the nose ( Fig. 1 and Video 1 ).

### NOSER Technique



**Video 2** Demonstration of step 1 of the nonsurgical elementary rhinoplasty (NOSER) technique: anterior nasal spine hyaluronic acid (HA) application.


**Video 3** Demonstration of step 2 of the nonsurgical elementary rhinoplasty (NOSER) technique: columella hyaluronic acid (HA) application.


**Video 4** Demonstration of step 3 of the nonsurgical elementary rhinoplasty (NOSER) technique: tip hyaluronic acid (HA) application.


**Video 5** Demonstration of step 4 of the nonsurgical elementary rhinoplasty (NOSER) technique: radix hyaluronic acid (HA) application.


**Video 6** Demonstration of step 5 of the nonsurgical elementary rhinoplasty (NOSER) technique: supratip hyaluronic acid (HA) application.

**Fig. 2:**
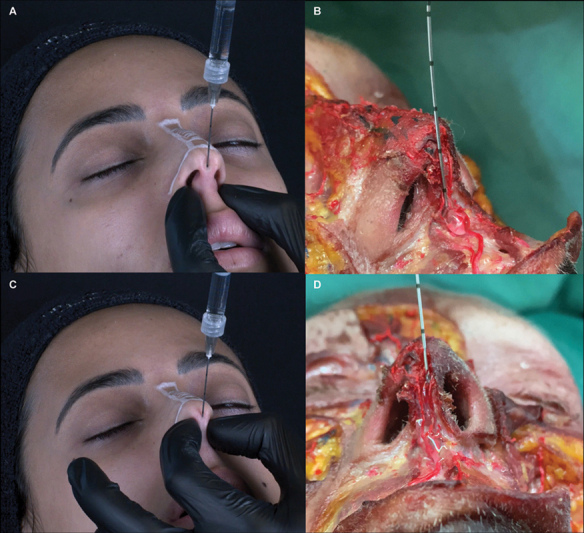
( **A** ) The nonsurgical elementary rhinoplasty (NOSER) technique. Step 1: deposition of HA supraperiosteal in the anterior nasal spine—0.1 mL. ( **B** ) Demonstration of the product bolus over the maxillary bone (transparent material) in a fresh frozen cadaver. ( **C** ) Step 2: column of HA, retroinjection interperichondrial—0.1 mL. ( **D** ) technique in a fresh frozen cadaver showing the cannula between the cartilages.

**Fig. 3:**
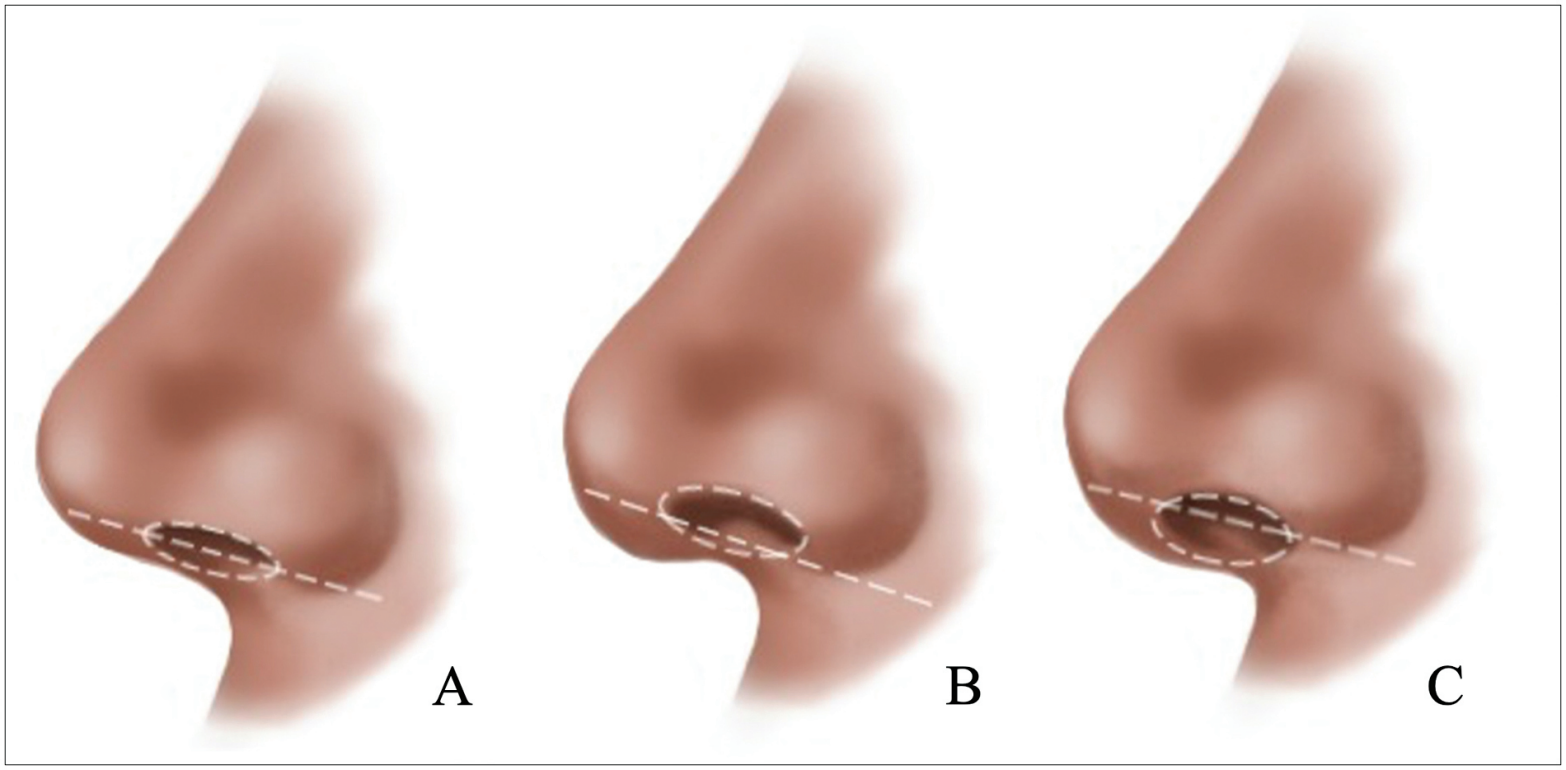
Types of columella: ( **A** ) normal, ( **B** ) retracted/hidden, and ( **C** ) pending/exposed.

**Fig. 4:**
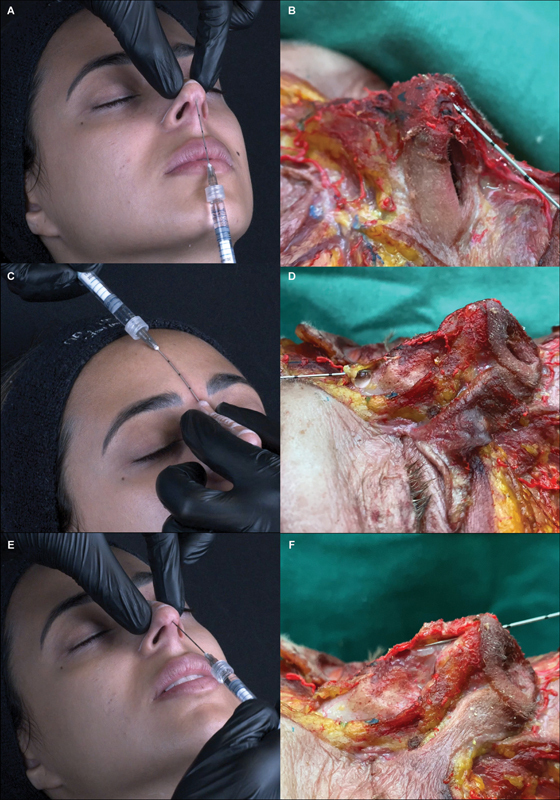
( **A** ) The nonsurgical elementary rhinoplasty (NOSER) technique. Step 3: small bolus injection into the nasal tip in the supraperichondrial plane (between the nasal domus—0.05 mL). ( **B** ) demonstration of the plan and product in fresh frozen cadaver, with the cannula positioned over the cartilages, not passing through them ( **C** ) Step 4: bolus application in the supraperiosteal plane in the radix (0.2 mL). ( **D** ) fresh frozen cadaver - cannula crosses the procerus muscle and touches the bone. ( **E** ) Step 5: microboluses application in the supraperichondrial plane in the supratip break (0.05 mL). ( **F** ) technique in a fresh frozen cadaver, depositing the product deeply.

- *Step 1: Anterior Nasal Spine* . Supports the columella, increases the columella labial angle (making it more obtuse), and structures the nasal tip. Through the lower entry point, the cannula is introduced between the medial ridges and goes down vertically with delicate rotation movements until it touches the maxillary bone (nasal spine). The cannula must go down parallel to the columellar line. Touching the bone, aspirate and inject a bolus of HA depending on the anterior nasal spine projection (overprojected [0.0 mL], average projection [0.1 mL], flattened [0.2 mL]). Pressure is laterally applied to the injection site to avoid migration of HA using the noninjecting hand, and the cannula opening hole is always be kept downward in all steps ( Fig. 2A, 2B and Video 2 ).
- *Step 2: Columella* . HA will act as a stem between the cartilages to increase support for the nasal tip and potentially correct possible columellar retraction. 6 The amount of HA applied will depend on the exposition of the columella. The perfect relationship between the columella and the nasal wing should not exceed 2 to 3 mm below a line parallel to the lateral view's lower margin of the nasal wing. Gunter et al propose a method for classifying the ideal pattern of the nasal wing-to-columella ratio. It is called retracted or hidden if the columella is positioned higher than the parallel line and pendulous or exposed if it is below this line. In our technique, we aim to restructure the columella body, respecting its exposure. Based on this, we may adjust the amount of HA as follows: pending/exposed (0.0 mL), normal (0.1 mL), hidden/retracted (0.2 mL; Fig. 3 ). Using a retroinjection technique via the interperichondrial layer, a column of filler is placed from the anterior spine of the maxilla till the inferior entry point ( Fig. 2C, 2D and Video 3 ).
- *Step 3: Tip* . HA will help project and define the tip. The cannula is introduced superiorly through the inferior orifice, passing over the domes of the inferior lateral nasal cartilages in the supraperichondrial layer. Avoid going through the cartilages due to the ligament that joins them. Apply two boluses of 0.05 mL per side (in each nose tip defining point) in cases of a more tapered tip or 0.05 to 0.1 mL centrally if the tip is bifid or wider. Press the entry point before removing the cannula to avoid the reflow ( Fig. 4A, 4B and Video 4 ).
- *Step 4: Radix.* The area between the nasion and the upper limit of the hump corresponds to the radix. HA intends to straighten the dorsum and correct the frontonasal angle. From the upper entry point, the cannula crosses the procerus muscle. Touch the bone and follow with the cannula through the nasal bone, scraping the supraperiosteal layer gently. Pinch the nose laterally to the aesthetic dorsal lines with the fingers of the noninjecting hand applying a light pressure against the bone to prevent lateral migration of the product and inject small and progressive boluses of 0.05 to 0.1 mL centrally for mild humps (<2 cm) or 0.2 to 0.3 mL for moderate humps. Repeat the injection and settle the product with a light massage until good aesthetic results, considering the nasofrontal angle (maximum total volume of 0.3 mL). For women, consider a more subtle, obtuse angle. To avoid a “Greek nose,” evaluate the results of the radix as you add filler to this area ( Fig. 4C, 4D and Video 5 ).
- *Step 5: Supratip* . The space between the inferior limit of the hump and the tip is the supratip. This step is indicated for patients in whom the supratip break is very accentuated or when desiring a straighter dorsum. Microboluses (total volume of 0.05–0.1 mL) in the supraperichondrial layer should be spread with the cannula facing down from the lower entry site. Avoid overcorrecting, reducing the fall of the tip risk, due to excess of HA ( Fig. 4E, 4F and Video 6 ).

**Fig. 5:**
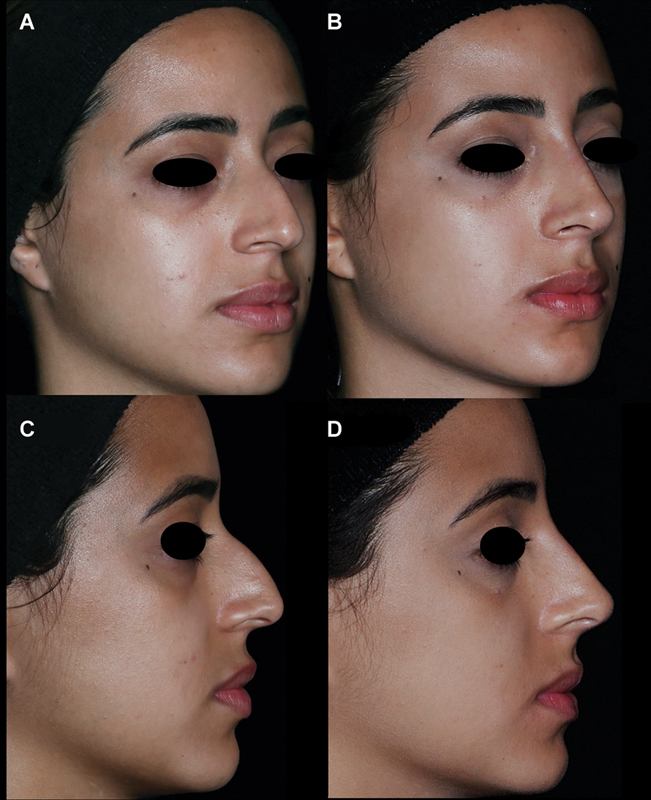
Before and after of a female patient treated using the nonsurgical elementary rhinoplasty (NOSER) technique - total 0.5 mL of HA. The maxillary crest and the columella with normal projections (steps 1 and 2 with 0.1 mL filler in each), thin and small tip (step 3 with 0.05 mL filler), sunk radix (step 4 with 0.2 mL filler), and desire for rectification of supratip break (step 5 with 0.05 mL filler). **A** ) 45 degree before ( **B** ) 45 degree after treatment ( **C** ) profile view before ( **D** ) profile result with NOSER technique. After 30 days of treatment we can observe a global upgrade in the nose, a projected and defined tip, straightened dorsum with hump disguise, redefined and parallel nasal aesthetic lines, improvement in the columelolabial and nasofrontal angles, and shortening of the nasal length.

Using the NOSER technique, we can achieve an important aesthetic improvement in the nose by widening the labial columellar angle, exposing the columella, projecting the tip, and aligning the dorsum, as shown in Fig. 5 . Reassessment is mandatory in 30 days for a possible touchup.

## Discussion

Nose remodeling with HA fillers is a suitable and noninvasive alternative to surgical rhinoplasty. 7 Over the last 3 years, we have observed that following all the stages of this technique, we may achieve delicate and natural results, reducing the chance of bruising and other problems. For this, some precautions are essential for preventing complications and/or unsightly results. (1) Keep the cannula parallel to the anterior nasal spine when injecting. If the cannula is inclined obliquely inward (toward the septum), there is a risk of HA accumulation in the nasal septum. Conversely, if it is angled obliquely outward, there is a chance of accumulation in the labial frenulum. (2) Check the frenulum after injection. The higher the amount of product placed, the greater the chance of local protrusion. In these cases, as the HA is important for local support, we recommend only gentle massage for accommodation.

Some patients have an exposed columella and a very acute (closed) labial columella angle. In these cases, we cannot inject into the entire columellar body to avoid worsening the exposure. A good alternative is to deposit a small amount of product (0.05–0.1 mL) with the cannula just at the base of the columella, without retroinjection, at the cephalometric point called the subnasal (transition from the cutaneous part of the lip to the columellar structure; Fig. 1B ). In this region, we were able to open the angle without weighing down the columella. In patients with an ideal and retracted columella, we continue with the injection as described.

We treat the radix before the supratip because often after straightening the dorsum the patient is already satisfied with the result and wants to maintain the supratip break. Furthermore, the skin in the radix region is less adherent to the deep planes and when filling this area, we can observe a slight lifting of the tip. Therefore, correction of the supratip as a refinement makes more sense than its correction before the radix. For those who, after the radix treatment, are still uncomfortable with the dorsum, treat the supratip as a final step to rectify it.

We can predict if the patient will have a “Greek nose.” Since nasal filling cannot reduce the hump, but only levels it with the dorsum, we draw an imaginary line from the highest point of the hump to the glabella. If this line goes beyond the nasion point (which delimits where the nose should begin), we will have a continuity of the frontal area to the nose and achieve that unsightly outcome. One option would be not to fill the radix until it is completely rectified.

We choose 22- to 23-gauge, 50-mm-long, cannulas based on scientific evidence that cannulas reduce the risk of complications as intravascular injections (ischemia, necrosis, blindness) and hematomas, but the main function of this technique is to systematize the injection sequence and guide appropriate volume placement in each nasal area for a beautiful, reproducible, and safe result. So, for expert injectors who feel more comfortable with a needle, the NOSER technique can be reproduced in the same way, just not using the cannula entry points and respecting the central region and the deep layers of the nose.

An important and interesting aspect of the technique is the possibility of its application in most types of noses. We do not need to perform all five steps or use the same amount of product volume on every patient. Individual assessment of patient needs is essential for unique results. For instance, a patient with a very projected maxillary crest and exposed columella does not need to receive the product in steps 1 and 2, benefiting only from other areas where some changes justify its injection.

Despite the safety and efficacy of this technique observed by the authors throughout the last 5 years, it is important to be aware of the training and handling of the injectors. They must have good knowledge of the anatomy, products, and injections to avoid traumas and/or lesions. Thus, training is crucial for using this technique. Furthermore, the use of cannulas rather than needles allows for more precise handling to access the deep plane (perichondrial and periosteal), while also reducing the points of trauma and bruising.

## Conclusion

The NOSER technique provides guidelines on filler volume per area and facilitates consistent nose remodeling. The NOSER technique aims to make rhinomodeling more accessible for experienced injectors, as it provides one of the most significant impacts on facial beautification and effectively contributes to patients' self-esteem.
